# Additive scales in degenerative disease - calculation of effect sizes and clinical judgment

**DOI:** 10.1186/1471-2288-11-169

**Published:** 2011-12-16

**Authors:** Matthias W Riepe, David Wilkinson, Hans Förstl, Andreas Brieden

**Affiliations:** 1 Department of Psychiatry and Psychotherapy II, Mental Health & Old Age Psychiatry, Ulm University, Ulm, Germany; 2 Department of Old Age Psychiatry, Southampton University, Southampton, UK; 3 Department of Psychiatry and Psychotherapy, Technische Universität München, München, Germany; 4 Department of Wirtschafts- und Organisationswissenschaften, Universität der Bundeswehr München, Neubiberg, Germany

**Keywords:** dementia, neurodegeneration, clinical studies, meta-analysis, effect sizes, Cohen's d

## Abstract

**Background:**

The therapeutic efficacy of an intervention is often assessed in clinical trials by scales measuring multiple diverse activities that are added to produce a cumulative global score. Medical communities and health care systems subsequently use these data to calculate pooled effect sizes to compare treatments. This is done because major doubt has been cast over the clinical relevance of statistically significant findings relying on *p *values with the potential to report chance findings. Hence in an aim to overcome this pooling the results of clinical studies into a meta-analyses with a statistical calculus has been assumed to be a more definitive way of deciding of efficacy.

**Methods:**

We simulate the therapeutic effects as measured with additive scales in patient cohorts with different disease severity and assess the limitations of an effect size calculation of additive scales which are proven mathematically.

**Results:**

We demonstrate that the major problem, which cannot be overcome by current numerical methods, is the complex nature and neurobiological foundation of clinical psychiatric endpoints in particular and additive scales in general. This is particularly relevant for endpoints used in dementia research. 'Cognition' is composed of functions such as memory, attention, orientation and many more. These individual functions decline in varied and non-linear ways. Here we demonstrate that with progressive diseases cumulative values from multidimensional scales are subject to distortion by the limitations of the additive scale. The non-linearity of the decline of function impedes the calculation of effect sizes based on cumulative values from these multidimensional scales.

**Conclusions:**

Statistical analysis needs to be guided by boundaries of the biological condition. Alternatively, we suggest a different approach avoiding the error imposed by over-analysis of cumulative global scores from additive scales.

## Background

Analysis of treatment efficacy is warranted to guarantee the quality of medical treatment and effective spending of resources. Across diseases, meta-analyses are assumed to be one the major tools to achieve this [1-4]. Meta-analyses are performed to come to an overall conclusion on clinical studies with different numerical results or using different assessment methods. One critical step in performing meta-analyses is to calculate the effect sizes for the studies to be included in the meta-analysis [5].

Degenerative diseases are of long duration and the diversity of their symptoms pose methodological difficulties not known in other fields of medicine: symptoms vary over time, fluctuate for random reasons, and may be replaced by new and different ones. To illustrate the reasoning on whether effect sizes and meta-analyses are suited to resolve the ambiguity of clinical study results in degenerative disease one of the most prevalent degenerative diseases, Alzheimer's disease (AD), will be used.

AD is the most frequent cause of dementia in old age and typifies the variability in clinical presentation and symptom changes over time that occurs in a degenerative disease. At onset of AD the medial temporal lobe is affected [6]. This results in the episodic memory deficit wihich is an early clinical hallmark of the disease [7]. As the disease spreads, other brain regions such as the frontal and parietal cortex are affected as well. The parietal cortex mediates activities such as spatial orientation and visuo-spatial functions [8,9]; the frontal cortex mediates executive functions, planning, attention, and working memory [10-12]. Spread of AD beyond the temporal lobe thus is characterized in functional terms by accruing deficits of spatial orientation, attention and executive functions as well as working memory and language [7]. This affliction of different brain regions and functions can be visualized using advanced imaging methods [13-15]. Despite an overall progress, symptoms may also fluctuate over the course of progressing dementia for random reasons. Apathy may turn to agitation which may disappear and followed by apathy, again. Regardless of this complexity, effect size calculation and meta-analyses of different studies use the addition of scores from many disparate functions to provide a global score for problems like 'cognition', 'behavior', or 'activities of daily living'. 'Cognition' comprises a multitude of activities such as episodic or working memory, attention, calculation, cognitive flexibility, praxis; 'behavior' comprises affect and emotion, delusion, agitation, irritability, and 'activities of daily living' comprise a wide variety of tasks for which the performance not only depends on the actual capabilities of the patient but also on her or his prior habits. Over the whole course of the disease, 'cognition' or 'behaviour' may be appropriate to assess overall dementia but over the time frame of clinical studies, usually one to two years, individual cognitive functions need to be focused on as the disease process over such short time spans is confined to specific functions and specific regions rather than the whole brain. At present, however, and for the last 30 years, clinical studies in AD have used global scales, i.e. multidimensional scales, to appraise the efficacy of interventions using instruments such as the Alzheimer's Disease Assessment Scale (ADAS) [16], the Mini-Mental-Status Examination (MMSE) [17], the Severe Impairment Battery (SIB) [18], the Neuropsychiatric Inventory (NPI) [19], the Katz activities of daily living scale (Katz-ADL) [20] amongst others.

Physicians and statisticians not well acquainted with the administration of neuropsychological tests neglect the impact of test difficulty on neurobiological associations. Task difficulty has a profound impact on the neural substrates engaged to solve the task. It was shown recently, that task difficulty is associated with recruitment of different neural patterns even in healthy subjects [21]. Thus, despite being similar activities, two tasks may rely on the integrity of different brain areas if the tasks vary in difficulty. Clearly then, the likelihood of maintaining performance on a specific task being measured with a particular instrument is dependent on disease severity and on time since diagnosis. The task may rely on different areas of the brain being recruited as degeneration reduces the relative amount of input from areas normally engaged in that function and showing a non-linear decline in dementia patients [22,23].

Multidemensional clinical scales combine different tasks, i.e. different activities, to assess overall severity of brain dysfunction. The cumulative score for these multidimensional scales results from summation of sub-scores representing specific activities. The relative contribution of the sub-scores to the total score, however, is variable, as is the task difficulty to assess specific activities in the different scales (e.g. the MMSE has a total score of 30 and scores 3 points for the recall of three words on single presentation and that task which is preserved till very late in the disease carries the same weight as the three points that could be obtained from recalling those words 5 minutes later a task that is very often one of the earliest signs of impairment, the ADAScog asks for recall of ten words on threefold presentation of the test and together with other memory items the function memory is represented with 27 points out of 70).

It was our goal to address the impact of non-linearity of disease progression and construction of multidimensional scales on the analysis of these additive global scales.

## Methods

### Basic model for the representation of function

Modeling the decline of function needs to reflect that tasks that are easy show a ceiling effect in assessment in early disease (i.e. the task is so easy or the underlying brain circuits are so insensitive to the disease process that the score does not decline over the initial time of the degenerative process) and in the later stages a floor effect (i.e. the task is so difficult or the underlying brain circuits are so severely affected from the disease process that the score is not sensitive enough to pick up further decline). Such a pattern was demonstrated for the items of the Mini-Mental-Status Examination [23,24], repeating of words is task with an early ceiling effect and delayed recall of memorized words is a task with an early floor effect. Accordingly we used an inverse exponential rule for modeling the decline of function with progressing disease: $f_{i}\left(t;a_{i},b_{i},c_{i}\right)={\left(a_{i}+b_{i}te^{c_{i}∕t}\right)}^{-1}$, where *i *= 1, 2, t_min _≤ *t *≤ *t*_max_, c_i _< 0.

Different *f*_i _represent different symptoms (e.g. memory, praxis, and so forth) declining over time according to parameters a_i_, b_i_, and c_i_, accessible by empirical studies, and t indicating time. Qualitatively, the arguments outlined below are also valid for various other functions than the inverse exponential function.

## Results

### Vulnerability and difficulty

**Figure 1:**
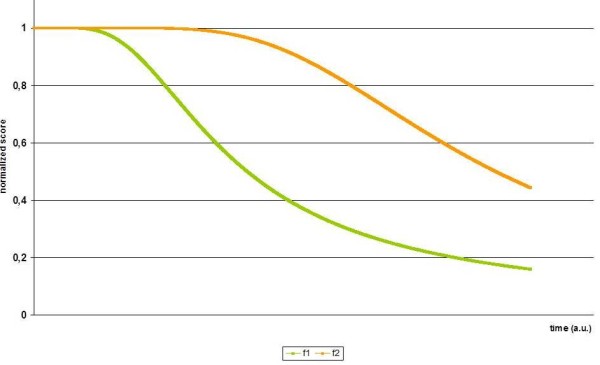
**Selective vulnerability and task difficulty**. $f_{i}\left(t;a_{i},b_{i},c_{i}\right)={\left(a_{i}+b_{i}te^{c_{i}∕t}\right)}^{-1}$, *i *= 1, 2, t_min _≤ *t *≤ *t*_max_,.For the orange curve the parameters of the formula in Figure 1 are: *a*_1 _= *b*_1 _= 1, *c*_1 _= -1/6. For the green curve the parameters are: *a*_2 _= *b*_2 _= 1, *c*_2 _= -1/20. The orange curve represents a symptom with a ceiling effect at the beginning of clinical disease (e.g. praxia in Alzheimer's disease), the green curve represents a symptom with a floor effect early during progression of disease (e.g. episodic memory in Alzheimer's disease).

Two examples for the decline of performance over time using the basic model are shown in Figure 1.

These curves can be interpreted in two different ways: I) function *f*_1 _and *f*_2 _represent different tasks, e.g. memory and praxis. In this interpretation, *f*_1 _represents an activity that early and rapidly declines with progression of disease (e.g. episodic memory in patients with Alzheimer's disease). The function *f*_2 _represents an activity that is upheld early during progression of disease with decline only occurring later (e.g. praxis in patients with Alzheimer's disease). Within this framework the neurobiological reason for the distinct time course of decline of function is selective vulnerability of brain regions. II) Alternatively, it may be assumed that the two curves represent the same task (e.g. spatial orientation). With this interpretation *f*_1 _represents measurement of the task with an instrument without a ceiling effect but with an early floor effect (e.g. spatial orientation in an unknown environment in patients with Alzheimer's Disease). The function *f*_2 _in this interpretation represents an instrument with an early ceiling effect and a late floor effect (e.g. spatial orientation in a known environment in patients with Alzheimer's Disease). In other words, *f*_1 _has a high task difficulty (reflecting disease progression or design of instrument) and *f*_2 _has a low task difficulty (reflecting disease progression or design of instrument).

### Multidimensional additive scales

We now assume two scales (e.g. the MMSE and the ADAScog), one scale represented by *F_A _*and another scale represented by *F_B_*, both comprised of two tasks following functions *f*_1 _(a task that declines early and rapidly over the course of disease) and *f*_2 _(a task that declines later during the course of disease) but weighted differently in *F_A _*and *F_B_*:

*F_j _(t; a_i_^,^, b_i_, c_i_, λ_ji_, i = *1, 2) = *λ*_*j*1_*f*_1_(t; *a*_1_*^,^, b*_1_, *c*_1_) + *λ*_*j*2_*f*_2 _(t; *a*_2_*^,^, b*_2_, *c*_2_) for *j *∈ {*A, B*} where *λ*_*j*1 _*λ*_*j*2 _with which the functions *f*_1 _and *f*_2 _are weighted in the scales *F_A _*and *F_B_*, respectively. Without loss of generality: *λ*_*j*1 _+ *λ*_*j*2 _= for *j *∈{*A, B*}.

To illustrate it: the cognitive part of the Alzheimer's Disease Assessment Scale (ADAScog) weights 'memory' with 27 out of 70 points: (word recall (max. 10), word recognition (max. 12), remembering test instructions (max. 5)). The Severe Impairment Battery (SIB) weights 'memory' with a maximum of 14 out of 100 points. The Mini Mental State Examination weights 'memory' with 6 out of 30 points. In contrast, 'orientation' is reflected in these scales with a maximum of 8 out of 70, 6 out of 100, and 10 out of 30, respectively.

**Figure 2:**
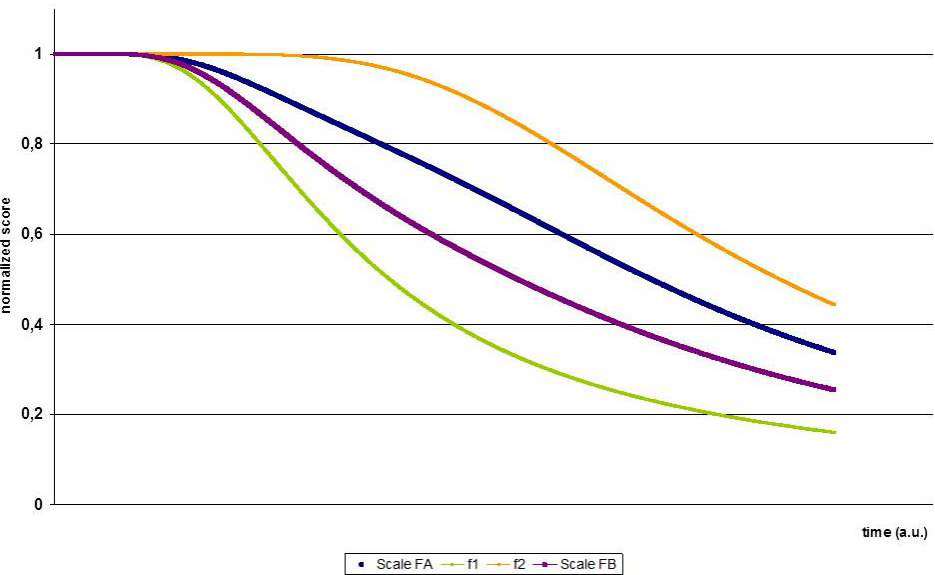
**Composite Scales**. Functions f_1 _and f_2 _as in Figure 1. Scale *F_A_: F_A_*(*t*; *a_i_*, *b_i_*, *c_i_*, *i *= 1, 2) = 3/8 *f*_1_(*t*; *a*_1_, *b*_1_, *c*_1_) + 5/8 *f*_2 _(*t*; *a*_2_, *b*_2_, *c*_2_). Scale *F_B_: F_B _*(*t*; *a_i_*, *b_i_*, *c_i_*, *i *= 1, 2) = 2/3 *f*_1_(*t*; *a*_1_, *b*_1_, *c*_1_) + 1/3 *f*_2 _(*t*; *a*_2_, *b*_2_, *c*_2_):. Hence, the scale *F_A _*is dominated by function f2 and scale *F_B _*is dominated by function f1. The graph shows normalized scores over time.

How combination of assessment of different tasks into one scale affects assessment of disease progression as measured with these scales is shown in Figure 2.

### Treatment effects

**Figure 3:**
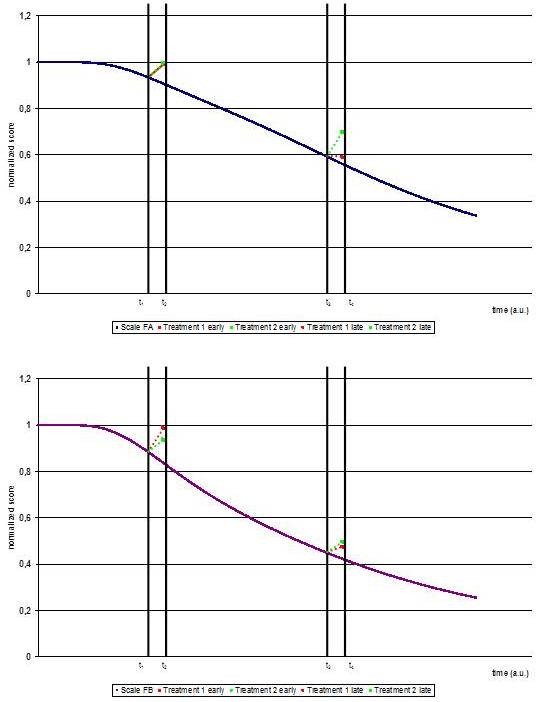
**Treatment effects**. Functions *f*_1 _and *f*_2 _and Scales *F_A _*and *F_B _*as in Figure 2. A treatment effect of 30% is assumed for *f*_1 _(Treatment 1) or *f*_2 _(Treatment 2). Upper panel) Effects on Scale *F_A _*at early and late time points. Lower panel) Effects on Scale *F_B _*at early and late time points. The graph shows normalized scores over time. The graph shows that the size of the treatment effect depends on the scale that is used.

We now assume treatment affects by scaling factors 1 + *δ*_i_, *i *= 1, 2, such that reflecting a purely symptomatic treatment effect on the progression of the disease for the treated group is described as (1 +*δ*_i _) *f_i _*(*t; a_i_, b_i_, c_i_*) for *i *= 1, 2, t_min _≤ *t *≤ *t*_max _Comparison of effect sizes or calculation of a common effect size in a meta-analysis naturally has to assume time-independence of the effect size - otherwise the result of bringing together results from multiple studies would strongly depend on how many studies with milder or more advanced severity of patients, respectively, are brought together in the analysis. The mathematical analysis below shows that a sufficient condition in the mathematical sense to achieve time independent effects is to assume that the standard deviation is proportional to the mean of the observed data. From a practical point of view this can be interpreted as a constant relative deviation. More precisely, Theorem 1 states that the effect size Cohen's *d *of both measurements is independent of the time of observation, i.e., *d*_*i*_(*t*) ≡ *d_i _*Hence, the necessary condition for applying for applying meta-analysis is satisfied. However, in general meta-analyses can also be performed with cumulative values of multidimensional scales and the question of time-independent effects have to be answered again. For this consider the additive scales *F_j _(t, a_i_, b_i_, c_i_, λ_ji_, i *= 1, 2) = *λ*_*j*1 _*f*_1_(*t*; *a*_1_, *b*_1_, *c*_1_) + *λ*_*j*2 _*f*_2_(*t*; *a*_2_*b*_2_*c*_2_) for *j *∈ {*A, B*} introduced before. Time-independence would follow if the effect sizes needs to be calculated in the intuitive way as *d_j_*(*t*) = *λ*_*j*1 _*d*_1_(*t*) + *λ*_*j*2 _*d*_2_(*t*). "Unfortunately", mathematical analysis (see below for more details) yields in that the effects size is a function depending on the weights *λ*_*j*1_, *λ*_*j*2 _*j *∈ {*A, B*} of the functions *f*_1 _and *f*_2 _in the composite scales *F_A _*and *F_B_*, the treatment effects *δ*_1_, *δ*_2_, and in contrast to the intuition in general on the functions *f_i_*, *i *= 1, 2, and - most important - the time t (Figure 3).

It is natural to ask, under which assumptions we can get rid of the general statement on time-dependence and still can guarantee time-independence for additive scales. The mathematical analyses shows that this is the case if we assume that over time the observed data are perfectly correlated with respect to the different scales and in addition if *δ*_1 _= *δ*_2 _(this means that the treatment effect is identical for both functions *f_i_, i *= 1, 2, representing different cognitive functions) or *λ_i _*= 0, *i *∈ {1, 2} The latter assumption means that function of interest is no longer multidimensional. Whether these assumption are either realistic or of relevant interest has to be decided in a preprocessing step.

However, in order to be able to calculate the time-dependent scaling factor in the general case, this would require to know the treatment effect on individual functions with given task difficulty and the exact weights of the individual functions in the composite scales as well as the time-dependency of the individual functions.

For example: a treatment effect of 30% improvement in function *f*_1 _or function *f*_2 _yields quite different effect sizes for early and late patients as assessed with scales *F_A _*or *F_B _*with results between 0.4624 and 0.6039 (Table 1).

**Table 1:** Calculation of effect sizes (Cohen's d) for early and late treatment as assessed with scale *F*_*A *_and *F*_*B*_.

	**Scale ***F_A _*	**Scale ***F_B _*
**Treatment 1 early**	0.4796	0.5693
**Treatment 1 late**	0.5736	0.6039
**Treatment 2 early**	0.5579	0.4624
**Treatment 2 late**	0,6005	0.5681

In scale *F_A_: F_A_*(*t*; *a_i_*, *b_i_*, *c_i_*, *i *= 1, 2) = 3/8 *f*_1_(*t*; *a*_1_, *b*_1_, *c*_1_) + 5/8 *f*_2 _(*t*; *a*_2_, *b*_2_, *c*_2_). In scale *F_B_: F_B _*(*t*; *a_i_*, *b_i_*, *c_i_*, *i *= 1, 2) = 2/3 *f*_1_(*t*; *a*_1_, *b*_1_, *c*_1_) + 1/3 *f*_2 _(*t*; *a*_2_, *b*_2_, *c*_2_). A treatment effect of 30% is assumed for *f*_1 _(Treatment 1) or *f*_2 _(Treatment 2).

### Inductive mathematical proof

If we assume the average progression of a disease with regard to two instruments within some specified period of time can be described by $f_{i}\left(t;a_{i},b_{i},c_{i}\right)={\left(a_{i}+b_{i}te^{c_{i}∕t}\right)}^{-1}$, *i *= 1, 2, t_min _≤*t *≤ *t*_max_, and that for any time *t *the underlying distribution of the random variable *X_i_*(*t*) is a normal one with mean *μ_i_*(*t*): = *f_i_*(*t*).

For its standard deviation *σ_i_*(*t*) we assume that always a percentage of 1- *α *of the distribution has a relative deviation from the mean from at most *β *percent. To be more precise, if *z*_*α*/2 _denotes the (1-*α*/2) -quantile of the standard normal distribution, then *σ_i_*(*t*) can be determined by the equations $1-\alpha =P\left(|\frac{X_{i}\left(t\right)-\mu_{i}\left(t\right)}{\sigma_{i}\left(t\right)}|\leq z_{\alpha /)2}\right)$

$=P\left(|X_{i}(t)-\mu_{i}(t)|\leq \underset{=\beta \mu_{i}(t)}{\underset{︸}{\sigma_{i}(t)\cdot z_{\alpha /2}}}\right),$ hence $\sigma_{i}\left(t\right)=\frac{\beta \mu_{i}\left(t\right)}{z_{\alpha /)2}}$,

whence for any time *t *we have$X_{i}\left(t\right)\sim N\left(\mu_{i}\left(t\right),\sigma_{i}^{2}\left(t\right)\right)=N\left(\mu_{i}\left(t\right),{\left(\beta \mu_{i}\left(t\right)/)z_{\alpha /)2}\right)}^{2}\right)$.

While the above models the case of untreated patients the effect of a proper medication is expressed by scaling factors 1 + *δ_i_, i = 1, 2*, i.e., on the average the progression of the disease for the treated group is described by (1 + *δ_i_*) *f_i_*(*t*; *a_i_*, *b_i_*, *c_i_*), *i *= 1, 2, t_min _≤ *t *≤ *t*_max_, where we assume like before that for any time *t *the random variable $X_{i}^{\delta_{i}}\left(t\right)$that describes the observed data at time *t *is again normally distributed with mean (1 + *δ_i_*) *μ_i_*(*t*) and, since the calculation of Cohen's *d *requires unchanged standard deviations, the same standard deviation like before, i.e., $\sigma_{i}\left(t\right)=\frac{\beta \mu_{i}\left(t\right)}{z_{\alpha /)2}}.$

Accepting the assumptions made above we obtain the following result for the effect size "Cohen's *d*" *d_i_*(*t*) of the treatment at time *t *for instrument *i, i = 1,2*.

### Theorem 1

The effect size Cohen's *d *is independent of the time of observation, i.e., *d_i_*(*t*) ≡ *d_i_*.

#### Proof 1

From the definition of Cohen's *d *we straightforward obtain

$$d_{i}\left(t\right)=\frac{\left(1+\delta_{i}\right)\mu_{i}\left(t\right)-\mu_{i}\left(t\right)}{\sigma_{i}\left(t\right)}=\frac{\delta_{i}\mu_{i}\left(t\right)}{\beta \mu_{i}\left(t\right)/)z_{\alpha /)2}}\equiv \frac{z_{\alpha /)2}\delta_{i}}{\beta }.$$

Next consider the case that we are interested in the composed function

$$f\left(t;a_{i},b_{i},c_{i},\lambda_{i},i=1,2\right)=\lambda_{1}f_{1}\left(t;a_{1},b_{1},c_{1}\right)+\lambda_{2}f_{2}\left(t;a_{2},b_{2},c_{2}\right),$$

where *λ*_1_, *λ*_2 _are non-negative scaling factors with, say, *λ*_1 _+ *λ*_2 _= 1. From an intuitive point of view we expect

$$d\left(t\right)=\lambda_{1}d_{1}\left(t\right)+\lambda_{2}d_{2}\left(t\right)\equiv \lambda_{1}\frac{z_{\alpha /)2}\delta_{1}}{\beta }+\lambda_{2}\frac{z_{\alpha /)2}\delta_{2}}{\beta }=\frac{z_{\alpha /)2}}{\beta }\left(\lambda_{1}\delta_{1}+\lambda_{2}\delta_{2}\right)$$

for the effect size *d*(*t*) of the composed scale. And in case our intuition is correct, time-independence as a desirable prerequisite for meta-analysis on, say, additive scales would immediately follow.

To compute *d*(*t*) for *f*(*t*; *a_i_*, *b_i_*, *c_i_*, *λ_i_*, *i *= 1, 2) we have to consider the random variable *X*(*t*) = *λ*_1_*X*_1_(*t*) + *λ*_2_*X*_2_(*t*) for untreated patients and $X^{\delta }\left(t\right)=\lambda_{1}X_{1}^{\delta_{1}}\left(t\right)+\lambda_{2}X_{2}^{\delta_{2}}\left(t\right)$for treated patients. Obviously, both variables are normally distributed with mean *μ*(*t*) = *λ*_1_*μ*_1_(*t*) + *λ*_2_*μ_2_*(*t*) and *μ^δ ^*(*t*) = *λ*_1_(1 + *δ*_1_)*μ*_1_(*t*) + *λ*_2 _(1 + *δ*_2_)*μ*_2_(*t*) respectively. For the variance *σ*^2^(t) of *X*(*t*) and hence by assumption also of *X^δ^*(*t*), we have the basic formula

$$\sigma^{2}\left(t\right)=\lambda_{1}^{2}\sigma_{1}^{2}\left(t\right)+2\lambda_{1}\lambda_{2}cor\left(X_{1}\left(t\right),X_{2}\left(t\right)\right)\sigma_{1}\left(t\right)\sigma_{2}\left(t\right)+\lambda_{2}^{2}\sigma_{2}^{2}\left(t\right),$$

where *cor *(*X*_1_(*t*), *X*_2_(*t*)) denotes the correlation of *X*_1_(*t*), and *X*_2 _(*t*),.

In the general case, i.e. without any restrictions on the correlation we obtain time-dependence on the effect size *d*(*t*) of the composed scale. To be more precise, we have

$$d\left(t\right)=\frac{\mu^{\delta }\left(t\right)-\mu \left(t\right)}{\sigma \left(t\right)}=\frac{\lambda_{1}\delta_{1}\mu_{1}\left(t\right)+\lambda_{2}\delta_{2}\mu_{2}\left(t\right)}{\sqrt{\lambda_{1}^{2}\sigma_{1}^{2}\left(t\right)+2\lambda_{1}\lambda_{2}cor\left(X_{1}\left(t\right),X_{2}\left(t\right)\right)\sigma_{1}\left(t\right)\sigma_{2}\left(t\right)+\lambda_{2}^{2}\sigma_{2}^{2}\left(t\right)}}.$$

To become more specific and to answer the question, whether time-independence can be guaranteed also for composed scales under special assumptions we consider as a simple example the case *cor *(*X*_1_(*t*), *X*_2_(*t*)) = 1 This assumption yields

$$\sigma^{2}\left(t\right)=\lambda_{1}^{2}\sigma_{1}^{2}\left(t\right)+2\lambda_{1}\lambda_{2}\sigma_{1}\left(t\right)\sigma_{2}\left(t\right)+\lambda_{2}^{2}\sigma_{2}^{2}\left(t\right)={\left(\lambda_{1}\sigma_{1}\left(t\right)+\lambda_{2}\sigma_{2}\left(t\right)\right)}^{2},$$

hence *σ*(*t*) = (*λ*_1_*σ*_1_(*t*) + *λ*_2_*σ*_2_(*t*)) and we can calculate Cohen's *d: *$d\left(t\right)=\frac{\mu^{\delta }\left(t\right)-\mu \left(t\right)}{\sigma \left(t\right)}=\frac{\lambda_{1}\left(1+\delta_{1}\right)\mu_{1}\left(t\right)+\lambda_{2}\left(1+\delta_{2}\right)\mu_{2}\left(t\right)-\left(\lambda_{1}\mu_{1}\left(t\right)+\lambda_{2}\mu_{2}\left(t\right)\right)}{\lambda_{1}\sigma_{1}\left(t\right)+\lambda_{2}\sigma_{2}\left(t\right)}=\frac{\lambda_{1}\delta_{1}\mu_{1}\left(t\right)+\lambda_{2}\delta_{2}\mu_{2}\left(t\right)}{\lambda_{1}\sigma_{1}\left(t\right)+\lambda_{2}\sigma_{2}\left(t\right)}.$ Using $\sigma_{i}\left(t\right)=\frac{\beta \mu_{i}\left(t\right)}{z_{\alpha /)2}}$we finally obtain$d\left(t\right)=\frac{z_{\alpha /)2}}{\beta }\cdot \frac{\lambda_{1}\delta_{1}\mu_{1}\left(t\right)+\lambda_{2}\delta_{2}\mu_{2}\left(t\right)}{\lambda_{1}\mu_{1}\left(t\right)+\lambda_{2}\mu_{2}\left(t\right)}$, which is in general still not independent of the time *t*.

In order to further analyze the dependence of the "composed Cohen's *d*" on the involved parameters we rewrite its formula. Under the assumption on standard deviations and correlation made above we obtain for the effect size:

### Theorem 2

$$d\left(t\right)=d\left(t,\lambda_{1},\lambda_{2},\delta_{1},\delta_{2}\right)=\frac{z_{\alpha /)2}}{\beta }\cdot \left(\delta_{1}+\left(\delta_{2}-\delta_{1}\right)\frac{1}{1+\left(\lambda_{1}\mu_{1}\left(t\right)/)\lambda_{2}\mu_{2}\left(t\right)\right)}\right).$$

#### Proof 2

We calculate

$$\begin{array}{ccc} d\left(t\right) & =\frac{z_{\alpha /)2}}{\beta }\cdot \frac{\lambda_{1}\delta_{1}\mu_{1}\left(t\right)+\lambda_{2}\delta_{2}\mu_{2}\left(t\right)}{\lambda_{1}\mu_{1}\left(t\right)+\lambda_{2}\mu_{2}\left(t\right)}=\frac{z_{\alpha /)2}}{\beta }\cdot \frac{\lambda_{1}\delta_{1}\mu_{1}\left(t\right)+\lambda_{2}\delta_{1}\mu_{2}\left(t\right)-\lambda_{2}\delta_{1}\mu_{2}\left(t\right)+\lambda_{2}\delta_{2}\mu_{2}\left(t\right)}{\lambda_{1}\mu_{1}\left(t\right)+\lambda_{2}\mu_{2}\left(t\right)} & \\ & =\frac{z_{\alpha /)2}}{\beta }\cdot \frac{\delta_{1}\left(\lambda_{1}\mu_{1}\left(t\right)+\lambda_{2}\mu_{2}\left(t\right)\right)+\left(\delta_{2}-\delta_{1}\right)\lambda_{2}\mu_{2}\left(t\right)}{\lambda_{1}\mu_{1}\left(t\right)+\lambda_{2}\mu_{2}\left(t\right)}\\ & =\frac{z_{\alpha /)2}}{\beta }\cdot \left(\delta_{1}+\left(\delta_{2}-\delta_{1}\right)\frac{\lambda_{2}\mu_{2}\left(t\right)}{\lambda_{1}\mu_{1}\left(t\right)+\lambda_{2}\mu_{2}\left(t\right)}\right)=\frac{z_{\alpha /)2}}{\beta }\cdot \left(\delta_{1}+\left(\delta_{2}-\delta_{1}\right)\frac{1}{1+\left(\lambda_{1}\mu_{1}\left(t\right)/)\lambda_{2}\mu_{2}\left(t\right)\right)}\right)\\ & =d\left(t,\lambda_{1},\lambda_{2},\delta_{1},\delta_{2}\right). \end{array}$$

From a theoretical point of view we can now observe the following:

1) If *δ*_1 _= *δ*_2_, then Cohen's *d *of the composed measure is independent of the time and in particular equals the weighted sum of the effect sizes *d*_1 _and *d*_2_, i.e.,

$$d\left(t\right)\equiv \frac{z_{\alpha /)2}}{\beta }\cdot \delta_{1}=\frac{z_{\alpha /)2}}{\beta }\cdot \delta_{1}\left(\lambda_{1}+\lambda_{2}\right)=\frac{z_{\alpha /)2}}{\beta }\left(\lambda_{1}\delta_{1}+\lambda_{2}\delta_{2}\right).$$

2) If *λ_i _*= 0, i ∈ {1, 2}, then Cohen's *d *of the composed measure is independent of the time, to be more precise $d\left(t\right)\equiv \frac{z_{\alpha /)2}}{\beta }\cdot \delta_{i}$. (Actually this reflects that the choice of parameter implies that the function of interest is no longer a composed one.)

The second observation straightforward leads to the question whether the choices of *λ_i _*= 0, i ∈{1, 2} are the extreme ones concerning *d*(*t*) over the domain D: = {*λ*: = (*λ*_1_, *λ*_2_)|*λ*_1_, *λ*_2 _≥ 0; *λ*_1 _+ *λ*_2 _= 1}?

### Theorem 3

$\frac{z_{\alpha /)2}}{\beta }\cdot \text{min}\left\{\delta_{1},\delta_{2}\right\}\leq \underset{\lambda \in D}{\text{min}}d\left(t,\lambda \right)\leq \underset{\lambda \in D}{\text{max}}d\left(t,\lambda \right)\leq \frac{z_{\alpha /)2}}{\beta }\cdot \text{max}\left\{\delta_{1},\delta_{2}\right\}.$

#### Proof 3

Without loss of generality assume that *δ*_1 _≤ *δ*_2_. Then it follows on the one side

$$\begin{array}{c} d\left(t\right)=\frac{z_{\alpha /)2}}{\beta }\cdot \left(\delta_{1}+\left(\delta_{2}-\delta_{1}\right)\frac{1}{1+\left(\lambda_{1}\mu_{1}\left(t\right)/)\lambda_{2}\mu_{2}\left(t\right)\right)}\right)\\ \leq \frac{z_{\alpha /)2}}{\beta }\cdot \left(\delta_{1}+\left(\delta_{2}-\delta_{1}\right)\right)=\frac{z_{\alpha /)2}}{\beta }\cdot \delta_{2}=\frac{z_{\alpha /)2}}{\beta }\cdot \text{max}\left\{\delta_{1},\delta_{2}\right\} \end{array}$$

and on the other side

$$d\left(t\right)=\frac{z_{\alpha /)2}}{\beta }\cdot \left(\delta_{1}+\left(\delta_{2}-\delta_{1}\right)\frac{1}{1+\left(\lambda_{1}\mu_{1}\left(t\right)/)\lambda_{2}\mu_{2}\left(t\right)\right)}\right)\geq \frac{z_{\alpha /)2}}{\beta }\cdot \delta_{1}=\frac{z_{\alpha /)2}}{\beta }\cdot \text{min}\left\{\delta_{1},\delta_{2}\right\}.$$

Note that we have always equality if *δ*_1 _= *δ*_2 _which reflects the first observation made above, hence scaling cannot change the effect size. However, if, say, *δ*_1 _<*δ*_2_, then Cohen's *d *can be changed by a factor of up to *δ*_2_/*δ*_1 _by choosing different scales.

Next let us consider the situation that either *δ*_1 _= 0 or *δ*_2 _= 0.

##### Corollary 1

Under the assumption made above on standard deviations and correlation we obtain for the effect size

$d\left(t\right)=\frac{d_{1}}{1+\left(\lambda_{2}\mu_{2}\left(t\right)/)\lambda_{1}\mu_{1}\left(t\right)\right)}\text{ if }\delta_{1}\neq 0=\delta_{2}\text{ and }d\left(t\right)=\frac{d_{2}}{1+\left(\lambda_{1}\mu_{1}\left(t\right)/)\lambda_{2}\mu_{2}\left(t\right)\right)}\text{ if }\delta_{1}=0\neq \delta_{2}.$

#### Proof

First note that$d_{i}\left(t\right)=\frac{z_{\alpha /)2}\delta_{i}}{\beta }$ is equivalent to $\frac{d_{i}\left(t\right)}{\delta_{i}}=\frac{z_{\alpha /)2}}{\beta }$. Hence, using Theorem 2 we obtain

$$d\left(t\right)=\frac{z_{\alpha /)2}}{\beta }\cdot \left(\delta_{1}+\left(\delta_{2}-\delta_{1}\right)\frac{1}{1+\left(\lambda_{1}\mu_{1}\left(t\right)/)\lambda_{2}\mu_{2}\left(t\right)\right)}\right)=\frac{d_{i}}{\delta_{i}}\cdot \left(\delta_{1}+\left(\delta_{2}-\delta_{1}\right)\frac{1}{1+\left(\lambda_{1}\mu_{1}\left(t\right)/)\lambda_{2}\mu_{2}\left(t\right)\right)}\right)$$

for *i *∈ {1, 2}.

If *δ*_1 _= 0 ≠ *δ*_2 _we conclude

$$\begin{array}{c} d\left(t\right)=\frac{z_{\alpha /)2}}{\beta }\cdot \left(\delta_{1}+\left(\delta_{2}-\delta_{1}\right)\frac{1}{1+\left(\lambda_{1}\mu_{1}\left(t\right)/)\lambda_{2}\mu_{2}\left(t\right)\right)}\right)=\frac{d_{2}}{\delta_{2}}\cdot \left(0+\left(\delta_{2}-0\right)\frac{1}{1+\left(\lambda_{1}\mu_{1}\left(t\right)/)\lambda_{2}\mu_{2}\left(t\right)\right)}\right)\;\\ =\frac{d_{2}}{1+\left(\lambda_{1}\mu_{1}\left(t\right)/)\lambda_{2}\mu_{2}\left(t\right)\right)}. \end{array}$$

If *δ*_1 _≠ 0 = *δ*_2 _we conclude

$$\begin{array}{c} d\left(t\right)=\frac{d_{1}}{\delta_{1}}\cdot \left(\delta_{1}-\delta_{1}\frac{1}{1+\left(\lambda_{1}\mu_{1}\left(t\right)/)\lambda_{2}\mu_{2}\left(t\right)\right)}\right)\;=d_{1}\left(1-\frac{1}{1+\left(\lambda_{1}\mu_{1}\left(t\right)/)\lambda_{2}\mu_{2}\left(t\right)\right)}\right)\\ =d_{1}\left(\frac{1+\left(\lambda_{1}\mu_{1}\left(t\right)/)\lambda_{2}\mu_{2}\left(t\right)\right)-1}{1+\left(\lambda_{1}\mu_{1}\left(t\right)/)\lambda_{2}\mu_{2}\left(t\right)\right)}\right)=\frac{d_{1}}{\left(\lambda_{2}\mu_{2}\left(t\right)/)\lambda_{1}\mu_{1}\left(t\right)\right)+1}. \end{array}$$

Finally let us compare in the situations *δ*_1 _= 0 or *δ*_2 _= 0 the composed Cohen's *d *with the intuitive choice *d*(*t*) = *λ_i_d_i_*.

##### Corollary 2

Under the assumption made above on standard deviations and correlation and assuming *μ*_1_(*t*) <*μ*_2_(*t*) für *t *∈ {*t*_min_, *t*_max_} we obtain for the effect size

$$d\left(t\right)<\lambda_{1}d_{1}\text{ if }\delta_{1}\neq 0=\delta_{2}\text{ and }d\left(t\right)>\lambda_{2}d_{2}\text{ if }\delta_{1}=0\neq \delta_{2}.$$

#### Proof

Using Corollary 1 for the case *δ*_1 _≠ 0 = *δ*_2 _we obtain

$$d\left(t\right)=\frac{d_{1}}{1+\left(\lambda_{2}\mu_{2}\left(t\right)/)\lambda_{1}\mu_{1}\left(t\right)\right)}\text{ = }\frac{\lambda_{1}\mu_{1}\left(t\right)}{\lambda_{1}\mu_{1}\left(t\right)+\lambda_{2}\mu_{2}\left(t\right)}d_{1}<\frac{\lambda_{1}\mu_{1}\left(t\right)}{\lambda_{1}\mu_{1}\left(t\right)+\lambda_{2}\mu_{1}\left(t\right)}d_{1}=\frac{\lambda_{1}}{\lambda_{1}+\lambda_{2}}d_{1}=\lambda_{1}d_{1}.$$

And in the case *δ*_1 _= 0 ≠ *δ*_2 _we obtain

$$d\left(t\right)=\frac{d_{1}}{1+\left(\lambda_{1}\mu_{1}\left(t\right)/)\lambda_{2}\mu_{2}\left(t\right)\right)}\text{ = }\frac{\lambda_{2}\mu_{2}\left(t\right)}{\lambda_{1}\mu_{1}\left(t\right)+\lambda_{2}\mu_{2}\left(t\right)}d_{1}>\frac{\lambda_{2}\mu_{2}\left(t\right)}{\lambda_{1}\mu_{2}\left(t\right)+\lambda_{2}\mu_{2}\left(t\right)}d_{1}=\frac{\lambda_{2}}{\lambda_{1}+\lambda_{2}}d_{1}=\lambda_{2}d_{1}.$$

## Discussion

Rather than drawing conclusions from clinical trials via the differences in the cumulative scores of clinical scales it has become a custom to calculate effect sizes. The intention being to allow comparison of the effect of treatments in the same indication but whilst using different instruments. Using meta-analytic procedures a pooled effect size then is calculated. Meta-analyses are assumed to be the tools to achieve an unbiased analysis of disease severity and the efficacy of treatments [1-4]. Meta-analyses thus are used to summarize results across studies and even across different indications. Considering the multitude of clinical trials and the multitude of treatments such methods are urgently needed and with certain study designs and endpoints this may be an appropriate procedure. It is one limitation of the present study that modulation of effect size calculation by instruments applied and disease stages analyzed applies only to additive scales. These, however, are used frequently in neurodegenerative disease and it is therefore necessary to be aware of the methodological boundary conditions for calculation of effect sizes for additive scales.

Simulation of decline of function in neurodegenerative disease with a non-linear representation of function demonstrates that calculation of effect sizes for early and late patients is subject to distortion by differences in the vulnerability of brain tissue or task difficulty and scale construction, respectively. Effect sizes are not inert to disease progression and the instruments used to detect it and therefore do not replace experienced clinical assessment of disease impact and treatment effect. Meta-analyses must not pool effect sizes from clinical trials in patients with different severity of disease. Clearly the use of the same scales across the whole disease process is not possible for reasons of differences in task difficulty creating floor and ceiling effects.

It has already been reported that the ADAS-cog and its subscales provide maximum information at moderate levels of cognitive dysfunction [25,26]. Raw score differences toward the lower and higher ends of the scale corresponded to large differences in cognitive dysfunction, whereas raw score differences toward the middle of the scale corresponded to smaller differences [25]. In more severe stages of dementia the ADAScog loses its sensitivity of change so much that the SIB was developed to assess patients who are unable to complete tests such as the ADAS-cog [18]. However, use of different composite scales is not possible since the subscales are not scaled according to task difficulty, are not balanced across different neuropsychological functions, and are weighted differently in different composite scales. A recent post-hoc analysis of published data is in good harmony with the conclusions from the simulation provided here and the mathematical analysis [27]. In that study [27] it was shown that effect size calculation is subject to an interaction of cognitive domain, disease severity, and instruments used for assessment.

In principle, these distortions by disease stage and treatments affecting different functions within a given scale could be measured and mathematical analysis (above and appendix) shows a way to estimate a scaling factor that needed to be introduced. Analysis of current shortcomings then needs to be extended. In the present model we only assume two functions representing two activities, which yields a scaling factor of up to *δ*_2_/*δ*_1 _(cf. above). Clinical scales such as the MMSE or the ADAScog are composed of a multitude of functions. When analyzing the ADAScog, for instance, at least four functions need to be considered: memory, orientation, language, and praxis. Therefore, in order to be able to estimate the relative scaling factors would require a very large population.

It has been suggested to call effect sizes of below 0.2 as 'small and above 0.5 as 'medium' [28]. The above analysis demonstrates that the naïve analysis of composite measures may bring about a false categorization of effect size. Effect size calculation of composite endpoints therefore cannot be used as a guideline for the judgment on therapeutic efficacy for neurobiological and statistical reasons. The numerical value of the analysis depends on the choice of the instrument and is subject to distortion by disease progression. Calculation of effect sizes, therefore, can not substitute for clinical assessment. Clinical expertise determines the choice of the instrument - the results therefore need to be interpreted with clinical expertise. Overall, statistical measures and meta-analyses of additive scales obfuscate, rather than clarify, the evidence on therapeutic efficacy in neurodegenerative disease.

In the past, clinical global assessments were the gold standard by which assessment scales were validated. In other words, scales were devised to act as a good proxy for clinical judgment which could be administered by less experienced clinicians. However, these scales clearly have great difficulties when extended over the range and time course of a degenerative disease. What may be a more satisfactory method of measuring change than combining many less than satisfactory study results would be to design a more sensitive way of capturing the clinical assessment. Clinical assessment uses parallel processing and multiple inputs which can account for variations in severity or even input of carepersons. Perhaps devising a more detailed global assessment with maybe 10 - 15 anchor points on a Likert scale that allows clinicians to provide a far more nuanced assessment than the present 7 (often then condensed to 5) point scale. For example it requires much greater evidence and confidence to move from minimal to major improvement than from no change to minimal improvement in most clinicians view and yet they represent similar degrees of improvement on the typical current global assessment scales. This tendency to conservative no change assessments caused by the lack of sensitivity of the scale may be why in the past the clinicians global assessment, whilst being the standard by which all patients in the real world and all other scales are assessed has not been regarded as a useful tool in clinical trials.

## Conclusions

In the face of the clear lack of credibility in pooling effect size calculations on grouped and yet disparate studies for meta-analysis it may be time to put the clinical appraisal that has served for generations back where it belongs as cornerstone of our efficacy assessments and decision making about the utility of treatments in neurodegenerative diseases.

## Competing interests

The authors declare that they have no competing interests.

## Authors' contributions

MWR, DW, and HF raised the ideas and elaborated the medical content. AB performed the mathematical proof. All authors read and approved the final manuscript.

## Pre-publication history

The pre-publication history for this paper can be accessed here:

http://www.biomedcentral.com/1471-2288/11/169/prepub
